# Omics for understanding synergistic action of validamycin A and *Trichoderma asperellum* GDFS1009 against maize sheath blight pathogen

**DOI:** 10.1038/srep40140

**Published:** 2017-01-06

**Authors:** Qiong Wu, Lida Zhang, Hai Xia, Chuanjin Yu, Kai Dou, Yaqian Li, Jie Chen

**Affiliations:** 1 School of Agriculture and Biology, Shanghai Jiao Tong University, 800 Dongchuan Road, Shanghai 200240, P. R. China; 2 State Key Laboratory of Microbial Metabolism, Shanghai Jiao Tong University, 800 Dongchuan Road, Shanghai 200240, P.R. China; 3 The Key laboratory of Urban (South) Agriculture, Ministry of Agriculture, Shanghai 200240, P.R. China

## Abstract

Sheath blight, causes by *Rhizoctonia* spp., threaten maize yield every year throughout the world. *Trichoderma* could degrade *Rhizoctonia solani* on maize mainly via competition and hyperparasitism, whereas validamycin A could efficiently inhibit the growth of *R. solani* via disturbing the energy system. By contrast, validamycin A is efficient but it takes effect in a short period, while *Trichoderma* takes effect in a long period though time-consuming. To overcome the disadvantages, *Trichoderma asperellum* GDFS1009 was used together with validamycin A. *In vitro* tests proved that the combined pathogen-inhibiting efficiency was significantly improved. Furthermore, results based on transcriptome and metabolome showed that validamycin A had no significant effects on growth, basic metabolism and main bio-control mechanisms of *T. asperellum* GDFS1009. Such few impacts may be attributed to detoxification and tolerance mechanism of *T. asperellum* GDFS1009. In addition, *T. asperellum* GDFS1009 has an ability to relieve the stress caused by validaymicn A. Meanwhile, liquid chromatography-mass spectrometry (LC-MS) results showed that only minor degradation (20%) of validamycin A was caused by *T. asperellum* GDFS1009 during cofermentation. All results together provide solid bases for validamycin A synergy with *T. asperellum* GDFS1009 in their combined biocontrol application.

Plant diseases play a direct role in the destruction of natural resources in agriculture. In particular, soil-borne pathogens cause important losses. *Rhizoctonia solani* and *Rhizoctonia zeae* are two major pathogens causing sheath blight in maize, which reduce quality and yield^1^,^2^.

As one of the most widely used biocontrol microbes, *T. asperellum* can inhibit and degrade pathogenic fungi by competition, hyperparasitism, and other antibiotic effects^3^,^4^,^5^,^6^,^7^. In fact, our previous study showed that *T. asperellum* grew faster than *R. solani* in a dual culture test. Therefore, *T. asperellum* can compete with pathogens for limited space and nutrients, which resulting in poor growth of pathogens at their interaction sites. Meanwhile, *T. asperellum* can secrete a series of CWDEs (Cell wall degrading enzymes) including chitinanse, β-1, 3-glucanase and protease, which can perform hyperparasitism action over *Rhizoctonia* mycelium, and further degrade it^8^,^9^,^10^,^11^. However, maize sheath blight, is a serious soil-borne disease, and the infection lasts until the late growth stage of maize plants, resulting in serious challenges for the effective biocontrol of the disease with *Trichoderma*^12^.

Validamycin A, an antibiotic, has long been used in China against maize sheath blight. It could be absorbed by *R. solani*, efficiently disturbs its highly depended energy system, trehalose pathway, and inhibits the growth of *R. solani*, but could not degrade it^13^,^14^. Furthermore, after years of use, as a widely used antibiotic, the control efficiency of validamycin A is somewhat declined, due to expected resistance of the pathogen to the chemical fungicide.

By contrast, validamycin A is efficient but it takes effect in a short period, while *T. asperellum* takes effect in a long period though time-consuming. To overcome the problems of *T. asperellum* and validamycin A, the combined use of validamycin A and *T. asperellum* are suggested as an alternative approach for improving efficacy against the pathogen^15^. Although there are very few strong evidences to support the combined use, it has already demonstrated that isolates of *T. asperellum* TK and TS can develop tolerance to chemical fungicides without decrease in antagonistic activity^16^. The prerequisite for the combined use of validamycin A and *Trichoderma* is that *Trichoderma* must have a high tolerance to validamycin A. To meet the requirement, the survival and biocontrol activities of *T. asperellum* have to be minimally impacted by validamycin A, and similarly, validamycin A should not be highly degraded or absorbed by *T. asperellum*. So far, it has no work conducted to unravel the interactive effects between *T. asperellum* and validamycin A on molecular basis.

So, in this study, we first clarified that *T. asperellum* GDFS1009 could be used in combination with validamycin A and their combined pathogen-inhibiting efficiency is significantly improved. Then we uncovered the molecular basis for the combined use at omics-wide (transcriptome and metabolome), physiology and biochemistry levels. To sum up, our study mainly focuses on: (1) the effect of validamycin A on *T. asperellum* GDFS1009 growth and biocontrol activities via some physiology experiments, including dual culture, optical and transmission electron microscope, enzyme activity detection, qRT-PCR and so on; (2) the global effect of validamycin A on *T. asperellum* GDFS1009’*s* transcriptome and metabolome based on RNA sequencing (RNA-seq) and gas chromatography-mass spectrometry (GC-MS) analyses, and the tolerance mechanism of *T. asperellum* GDFS1009 against validamycin A; (3) the detection of validamycin A degradation and absorption by *T. asperellum* GDFS1009 by liquid chromatography-mass spectrometry (LC-MS) analysis. These studies comprehensively illustrated the synergistic mechanism by which validamycin A and *T. asperellum* used against maize sheath blight pathogen infection.

## Results

### The synergistic antibiotic effect of validamycin A and *T. asperellum* GDFS1009 on sheath blight

After incubation for 1 d, validamycin A could be efficiently absorbed by *R. solani (R. zeae*) and its bacteriostasis was highly significant. At this stage, the colony area of *R. solani (R. zeae*) was barely 222.34 ± 5.29 mm^2^ (357.14 ± 7.25 mm^2^) in the validamycin A experimental group, while it reached 2544.94 ± 92.13 mm^2^ (1893.21 ± 9.55 mm^2^) in the blank control group. Though *T. asperellum* GDFS1009 which grew rapidly had a relatively obvious bacteriostatic efficacy, its impact on bacteriostasis was weaker than that of validamycin A. After *T. asperellum* GDFS1009 experimental treatment, the colony area of *R. solani (R. zeae*) was 1699.33 ± 14.39 mm^2^ (2153.05 ± 62.11 mm^2^). Furthermore, the synergistic effect of validamycin A combined with *T. asperellum* GDFS1009 was not present at this stage. The colony area of *R. solani (R. zeae*) was barely 221.17 ± 11.06 mm^2^ (335.92 ± 35.63 mm^2^) in the experiment with validamycin A combined with *T. asperellum* GDFS1009, which demonstrated no significant difference compared with validamycin A experimental treatment alone.

**Figure 1:**
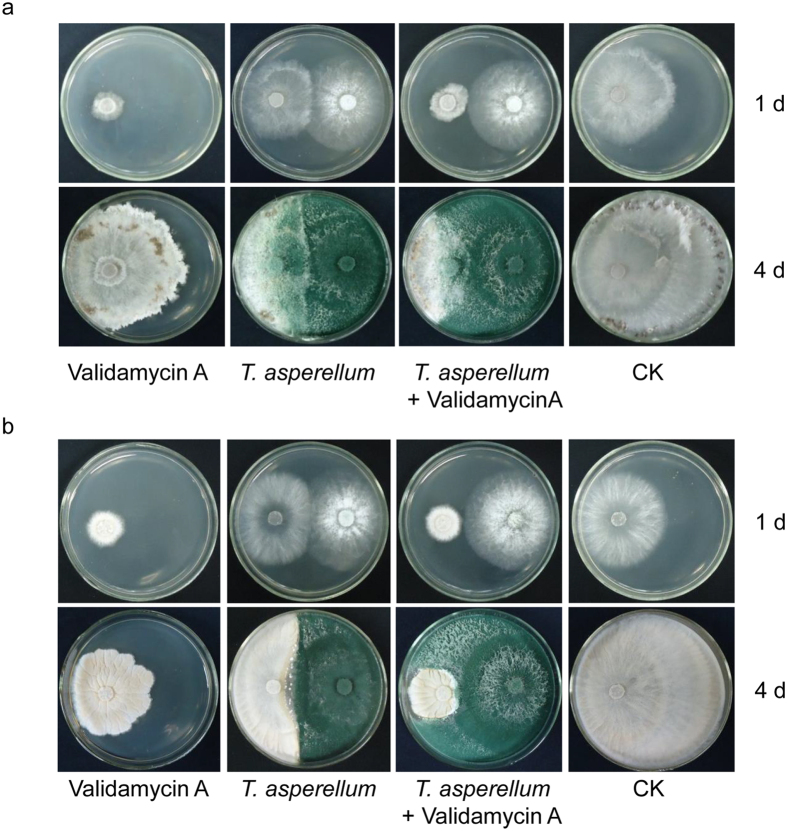
Synergistic effect of validamycin A and *T. asperellum* GDFS1009 on sheath blight. (**a**) Synergistic effect on *R. solani* after cultivated 1d and 4 d; (**b**) Synergistic effect on *R. zeae* after cultivated 1d and 4 d.

However, with increased experimental time, the bacteriostasis of validamycin A began to attenuate and gradually removed. After incubation for 4 d, the colony area of *R. solani (R. zeae*) increased to 3956.08 ± 70.52 mm^2^ (1912.26 ± 102.48 mm^2^) in the validamycin A experimental group, while it was 6351.89 ± 40.97 mm^2^ (6352.66 ± 40.95 mm^2^) in the blank control group. Nevertheless the advantage of biological containment of *T. asperellum* GDFS1009 was represented at this stage, and the colony area of highly inhibited *R. solani (R. zeae*) decreased to merely 1565.78 ± 46.53 mm^2^ (2310.5 2 ± 107.04 mm^2^). Furthermore, the synergistic effect of validamycin A combined with *T. asperellum* GDFS1009 was reflected. Under successive impacts of antibiosis, competition, and hyperparasitism, the colony diameter of *R. solani (R. zeae*) was 1058.46 ± 68.08 mm^2^ (724.16 ± 17.28 mm^2^), significantly less than any single factor treatment (Fig. 1 and Supplementary Figure S1).

### Global impact of validamycin A on *T. asperellum* GDFS1009 based on RNA-seq and GC-MS data

From 24 h and 48 h cultures of *T. asperellum* GDFS1009, 11,845 genes and 11,801 genes were obtained, respectively. After treatment with validamycin A for 24 h and 48 h, the differentially expressed genes in *T. asperellum* GDFS1009 were 8.93% and 6.78%, respectively, when compared with the control sample (Supplementary Figure S2a and b).

By GC-MS analysis of 24 h *T. asperellum* GDFS1009 cultured samples, 104 types of compounds were obtained, including 18 amino acids and their precursors, 15 types of amines, 15 types of carbohydrates, 10 types of fatty acids, 15 types of organic acids, 8 types of phosphate, and 23 compounds from other categories (Supplementary Figure S2c). Comprehensive analysis showed that, compared with the control sample, the sample after validamycin A treated had only five compounds with significant changed, which were: fatty acids, carbohydrates, amino acids, and amines. Their proportion was very small, only 4.81%.

### Effect of validamycin A on growth and competition of *T. asperellum* GDFS1009

**Figure 2:**
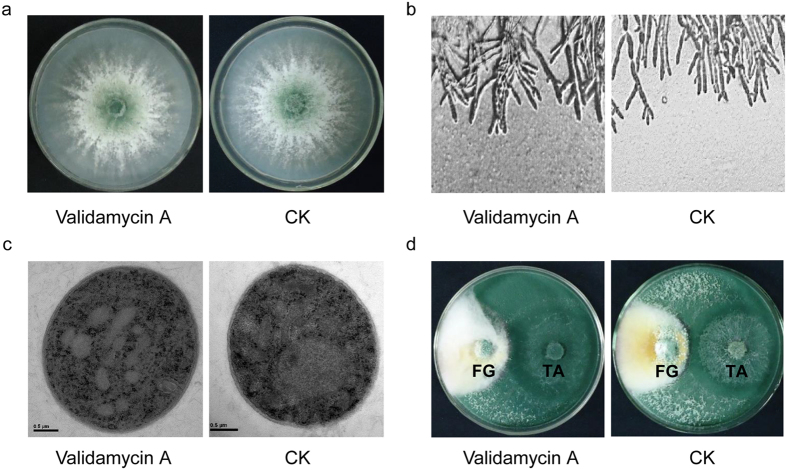
Impact of validamycin A on growth, morphology, ultrastructure, and competition of *T. asperellum* GDFS1009. (**a**) Mycelium growth; (**b**) Mycelium morphology under optical microscope; (**c**) Mycelium ultrastructure under transmission electron microscope; (**d**) Competition to *F. graminearum*. FG: *F. graminearum*; TA: *T. asperellum* GDFS1009.

In 4 d of culture, compared with the control, the colony size, mycelium morphology and mycelium ultrastructure were not changed in *T. asperellum* GDFS1009 treated with validamycin A (Fig. 2a,b,c).

After treated with validamycin A for 4 d, *T. asperellum* GDFS1009 displayed the same level of competition on *Fusarium graminearum* (Fig. 2d).

**Figure 3:**
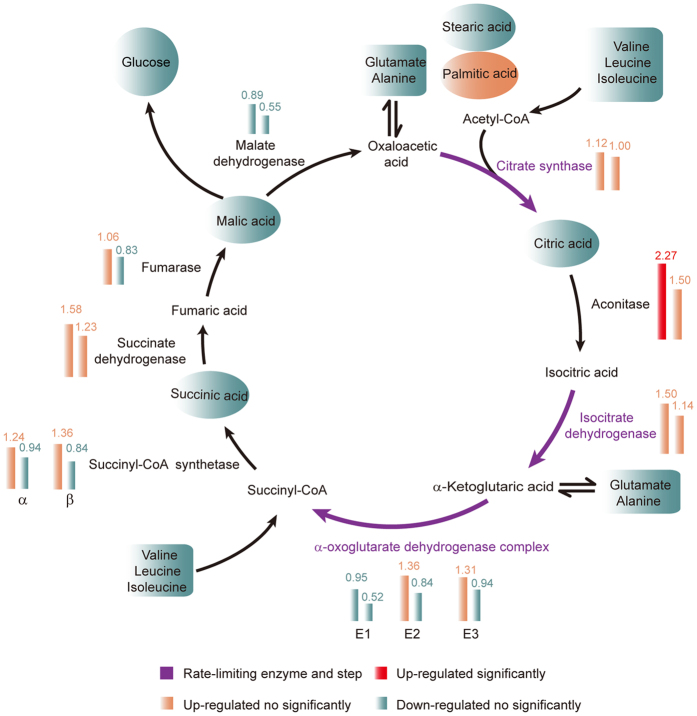
Impact of validamycin A on TCA cycle and related carbohydrate, fatty acid and amino acid metabolism based on RNA-seq and GC-MS. Column diagram represents the RNA-seq results at 24 h and 48 h, while oval and tetragonum represent the GC-MS results at 24 h. Red represents significantly up-regulated, light pink represents up-regulated no significantly, and light blue represents down-regulated no significantly. Purple represents rate-limiting step.

As the tricarboxylic acid cycle (TCA) cycle is the basis for biological metabolism and is the hub of carbohydrate, fatty acid, and amino acid metabolism pathways^17^,^18^, which is related to growth and competition of *T. asperellum* GDFS1009, corresponding RNA-seq data of 24 h and 48 h cultures was analyzed and the results showed that, compared to the control *T. asperellum* GDFS1009, *T. asperellum* GDFS1009 treated with validamycin A had only increased aconitase, but not the rate-limiting enzymes. GC-MS metabolomics 24 h data analysis showed that, in *T. asperellum* GDFS1009 treated with validamycin A and control, the yield of citric acid, succinate, and malic acid was not significantly different. Comprehensive analysis of RNA-seq and GC-MS results showed that, validamycin A had no significant effect on the TCA cycle in *T. asperellum* GDFS1009 (Fig. 3 and Supplementary Tables S2–S4).

RNA-seq results showed that, in *T. asperellum* GDFS1009 treated with validamycin A for 24 h, in 13 amino acid metabolic pathways, a total of 72 genes were up-regulated and 11 were down-regulated. After treatment for 48 h, in 12 amino acid metabolic pathways, a total of 28 genes were up-regulated, and 2 genes were decreased (Supplementary Table S3). GC-MS metabolomics data of 24 h showed that, there is no significant difference in the yield of these amino acids between the control and validamycin A treated *T. asperellum* GDFS1009 (Supplementary Table S5). Comprehensive analysis of the results of gene expression profiles and GC-MS metabolomics showed that, validamycin A had no significant effect on the synthesis and degradation pathways of amino acids in *T. asperellum* GDFS1009. In addition, RNA-seq results showed that, few genes were changed in fatty acid metabolic pathways (Supplementary Table S3). GC-MS results showed that, there is no significant difference in yields of these fatty acids between control *T. asperellum* GDFS1009 and *T. asperellum* GDFS1009 treated with validamycin A (Supplementary Table S6). There is also no significant difference in yields of glucose between control *T. asperellum* GDFS1009 and *T. asperellum* GDFS1009 treated with validamycin A (Supplementary Table S7). Comprehensive analysis of the above RNA-seq and GC-MS results showed that, validamycin A had no significant effect on the TCA cycle and related carbohydrates, lipids, and amino acid metabolism in *T. asperellum* GDFS1009, namely validamycin A does not affect the basal metabolic pathways of *T. asperellum* GDFS1009.

### Effect of validamycin A on the hyperparasitism of *T. asperellum* GDFS1009

**Figure 4:**
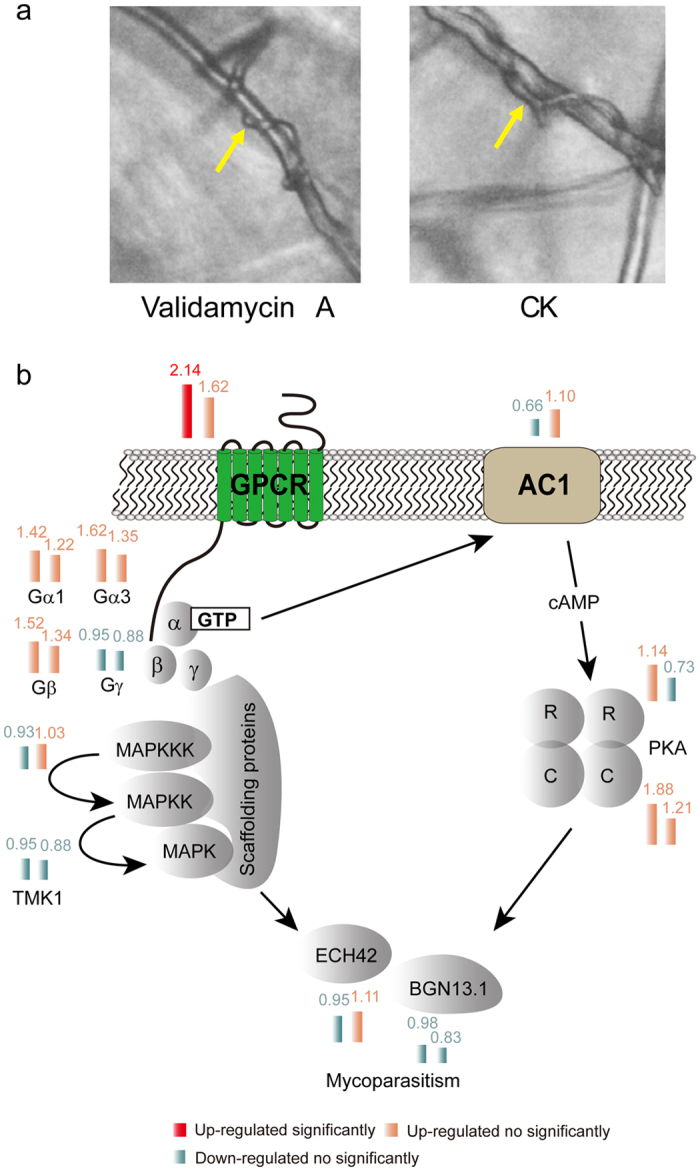
Impact of validamycin A on hyperparasitism of *T. asperellum* GDFS1009. (**a**) Hyperparasitism of *T. asperellum* GDFS1009 on *F. graminearum*; (**b**) Impact of validamycin A on G protein-MAPK-cAMP pathway of *T. asperellum* GDFS1009 based on RNA-seq. Column diagram represents the RNA-seq results at 24 h and 48 h. Red represents significantly up-regulated, light pink represents up-regulated no significantly, and light blue represents down-regulated no significantly.

Results of optical and scanning electron microscopy showed that after treatment with validamycin A, *T. asperellum* GDFS1009 displayed the same level of hyperparasitism on *F. graminearum* (Fig. 4a). During fermentation for 3–4 d, the chitinase production of *T. asperellum* GDFS1009 reached a plateau. The enzyme activity in the *T. asperellum* GDFS1009 treated with validamycin A at 4 d fermentation is 121.48 ± 9.11 U/mL, compared with 119.84 ± 6.23 U/mL in control, showing that validamycin A had no effect on the production of *T. asperellum* GDFS1009 chitinase (Supplementary Figure S3a). Meanwhile, validamycin A had no influence on cellulase production in *T. asperellum* GDFS1009. After fermentation of 3 d, the enzyme activity reached the maximum, the enzyme activity after validamycin A treatment was: 1.717 ± 0.212 U/mL, compared with 1.72 ± 0.11 U/mL in control (Supplementary Figure S3b).

In *Trichoderma*, G protein-MAPK (Mitogen-activated protein kinases)-cAMP (protein kinase A system) signaling pathway is associated with hyperparasitism^19^,^20^. After treatment with validamycin A at 24 h, RNA-seq data showed that only G-protein gene was significantly up-regulated, while, AC, PKA, TMK1, ECH42 genes and so on in G protein-MAPK-cAMP signaling pathway were not significantly changed. Then, at 48 h, all the genes were not significantly changed (Fig. 4b and Supplementary Table S8). Furthermore, qRT-PCR validation showed conformably results (Supplementary Figure S3c and d). Therefore, we may infer that validamycin A does not affect the hyperparasitism of *T. asperellum* GDFS1009.

### The degradation and absorption rate of validamycin A by *T. asperellum* GDFS1009

**Figure 5:**
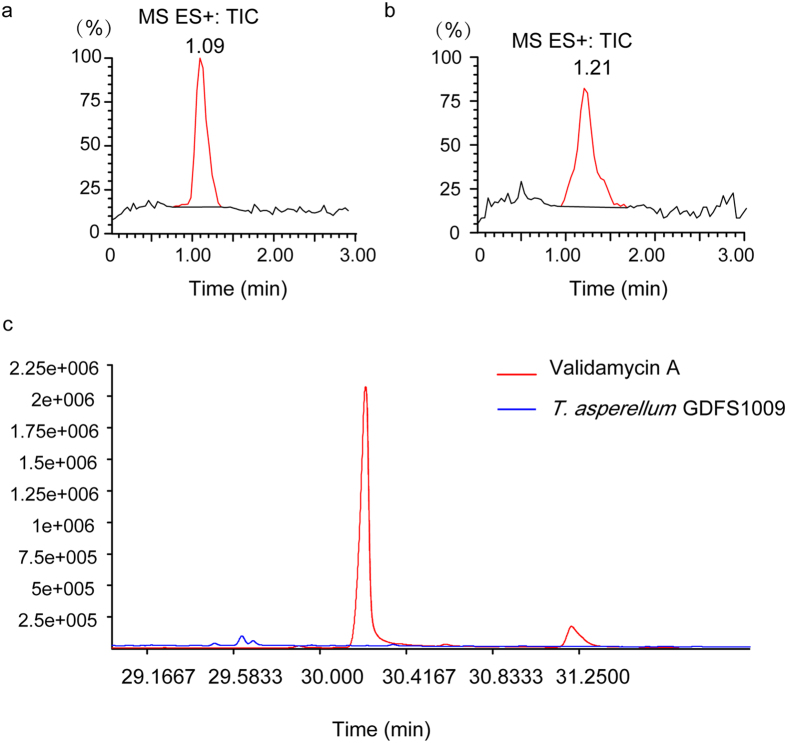
Impact of *T. asperellum* GDFS1009 on validamycin A. (**a**) validamycin A standards in fermentation broth; (**b**) Detection of validamycin A in fermentation broth after co-cultured 7 d with *T. asperellum* GDFS1009 via LC-MS; (**c**) Detection of validamycin A in *T. asperellum* GDFS1009 after co-cultured 4 d via GC-TOF-MS.

LC-MS results showed that, co-fermentation of *T. asperellum* GDFS1009 and validamycin A in PD broth for 7 d, resulted in a limited 20% degradation of validamycin A, indicating that *T. asperellum* GDFS1009 has no significant effect on validamycin A (Fig. 5a and b). Additionally, gas chromatography/time-of-flight mass spectrometry (GC-TOF-MS) results showed that no validamycin A was detected in the mycelium of *T. asperellum* GDFS1009 (Fig. 5c) exposed to validamycin A.

### Transient impacts of validamycin A on *T. asperellum* GDFS1009 detoxification genes

Peroxidase, monooxygenase, glutathione transferase, catalase, and ABC translocatorare related to biological detoxification and stress^21^,^22^. After 24 h treatment with validamycin A, 8 peroxidase genes, 6 monooxygenase genes, 1 superoxide dismutase gene and 2 catalase genes in *T. asperellum* GDFS1009 were significantly up-regulated, but none of which were still significantly up-regulated at 48 h. Additionally, 3 glutathione S-transferase genes, 1 thioredoxin gene and 6 ABC translocator genes in *T. asperellum* GDFS1009 were significantly up-regulated at 24 h, while at 48 h only 1 glutathione S-transferase gene, 1 thioredoxin genes and 4 ABC translocator genes were still significantly up-regulated. It is notable that there were 3 ABC translocator genes significantly down-regulated (Table 1).

## Discussion

*T. asperellum* GDFS1009 could inhibit *R. solani* on maize mainly via competition and hyperparasitism, and further degrade it, whereas validamycin A, as a widely used antibiotic, could be absorbed by *R. solani*, efficiently disturbs its highly depended energy system, trehalose pathway, and inhibits the growth of *R. solani*, but could not degrade it^3^,^4^,^5^,^6^,^7^,^13^. By contrast, validamycin A is efficient but it takes effect in a short period, while *T. asperellum* GDFS1009 takes effect in a long period though time-consuming. It was proven that, as compared to a single agent, when validamycin A and *T. asperellum* GDFS1009 are used together and complementary advantages, the control effect on sheath blight was significantly enhanced (Fig. 1). Similarly, it is showed that *T. harzianum* and *T. asperelloides* can develop tolerance to fungicides without compromising their biological activity (growth and parasitism against *Fusarium*)^23^.

However, this is the first detailed report on the possible effects of validamycin A on *Trichoderma*. The results showed that, validamycin A had no obvious effect on growth, mycelium morphology, mycelium ultrastructure and the effect of competition of *T. asperellum* GDFS1009 (Fig. 2). In consideration of primary metabolism which is the energy basis for *Trichoderma* growth and competition, we used two approaches of metabolomics and transcriptomics to globally explore the sensitive metabolism pathway upon validamycin A interaction. Comprehensive analysis suggested that there were no obvious effect on its TCA cycle, glycolytic pathway, lipid and amino acid metabolism (Fig. 3 and Supplementary Tables S2–S7). To sum up, validamycin A does not affect the function of competition of *T. asperellum* GDFS1009.

In *Trichoderma*, G protein-MAPK-cAMP signaling pathway is associated with hyperparasitism^19^,^20^. MAPK gene *tmk1* in *T. atroviride* is an important signal gene in hyperparasitism. After knockout of *tmk1*, the hyperparasitic capacity of *T. atroviride* was decreased, while the expressions of chitinase gene *nag1* and *ech42* were significantly induced. In this study, as the hyperparasitism related genes were analyzed via RNA-seq and qRT-PCR, together with dual culture tests, we could infer that validamycin A had no significant effect on hyperparasitism of *T. asperellum* GDFS1009 (Figs 2d and 4 and Supplementary Tables S8).

Meanwhile, LC-MS analysis showed that the absorption and degradation of validamycin A by *T. asperellum* GDFS1009 was limited (Fig. 5). These researches provided a strong theoretical basis for synergistic effect of validamycin A and *T. asperellum* GDFS1009, and also provide a theoretical basis for combined use via more modes (for example, validamycin A with biocontrol factors or spores granules of *T. asperellum* GDFS1009).

In order to uncover the co-exist mechanism of validamycin A and *T. asperellum* GDFS1009, detoxification and tolerance related genes should be analyzed. It is reported that peroxidase, monooxygenase, glutathione transferase, and catalase are related to biological detoxification and stress. Invasion of external toxic substances could stimulate fungal overexpression of a large number of enzymes, including monooxygenase and peroxidase, which could produce a lot of reactive oxygen species (ROS). Then, toxic substances could be oxidized, form toxic substances-glutathione (GSH) complex via glutathione transferase, and be eliminated from the fungi by catalase etc^21^. After 24 h treatment with validamycin A, lots of related genes in *T. asperellum* GDFS1009 were significantly up regulated, almost all of which were down to normal levels at 48 h (Table 1). It is explained that the ability of *Trichoderma* to withstand relatively high concentrations of a variety of synthetic and natural toxic compounds, including its own antibiotics, depends on efficient cell detoxification mechanisms supported by a complex system of membrane pumps^24^. It is well known that the genome of *Trichoderma* includes ABC transporters (ATP binding cassette (ABC) transporters), which are members of a protein superfamily that efflux drugs from cells of target organisms. Thus transporters may provide a mechanism of protection against cytotoxic drugs and xenobiotic agents. The natural function of ABC transporters in plant pathogenic fungi may relate to transport of plant-defense compounds or fungal pathogenicity factors. The ABC transporters may explain the natural tolerance of *Trichoderma* to fungicides, and their ability to successfully survive in extreme environments^22^. After 24 h treatment with validamycin A, 6 ABC transporter genes were significantly up-regulated, while after 48 h treatment, 4 ABC transporter genes remains significantly up-regulated expression, but there are 3 ABC transporter genes appearing significantly down regulation (Table 1). According to all the results, we speculated that validamycin A may have a slight stimulation on *T. asperellum* GDFS1009 but without toxicity. Within 24 h, *T. asperellum* GDFS1009 spores began germination, and the mycelium is immature. It is more sensitive to external stimuli, so the expression of some genes related to detoxification and stress tolerance were significantly increased; at 48 h, the mycelium is matured and prosperous, and gradually adapts to external stimuli, so the expression of these detoxification genes were decreased.

It is already known that the trehalose pathway in sheath blight pathogen (*Rhizoctonia* spp.) is a target of validamycin A attacks^13^. Validamycin A could be absorbed in abundance by *R. solani*, efficiently disturbs its highly depended energy system, trehalose pathway, impacts the glucose production and inhibits the growth of *R. solani*. But in our study, the glucose production was not impacted in *T. asperellum* GDFS1009 (Supplementary Table S7), and the growth of *T. asperellum* GDFS1009 was not impacted by validamycin A (Fig. 2a,b and c), indicating that the trehalose pathway in *T. asperellum* GDFS1009 is not the target of validamycin A, which is distinguished to *R. solani*. This may also be the reason why validamycin A and *T. asperellum* GDFS1009 could co-exist together.

It is notable that this study was simply performed at the primary metabolism level to understand the impact of validamycin A on *T. asperellum* GDFS1009, so the influence at the secondary metabolism level cannot be explained. Whereas, based on our findings from culture tests, we assumed a certain impact of validamycin A over *T. asperellum* GDFS1009 secondary metabolism, if it did happen, the slight changes of secondary metabolites may not associate with pathogen antagonistic activity since not all secondary metabolites are inhibitory to sheath blight pathogen.

At present we cannot confirm the impact of validamycin A on *T. asperellum* GDFS1009 *in vivo* since most of the experiments were completed *in vitro* which cannot reflect the whole situation of validamycin A-*Trichoderma* in its natural condition. Therefore, our next study will evaluate what happens to the interaction of *T. asperellum*-validamycin A in the presence of pathogen and maize plants.

## Methods

### Fungal strains

The biocontrol fungus *T. asperellum* GDFS1009 was preserved in China General Microbiological Culture Collection Center (CGMCC NO. 9512), Beijing, China. *R. solani, R. zeae* and *F. graminearum* on maize were used as target pathogens for examination of biocontrol effects.

### Analysis of the effect of synergetic inhibition of *T. asperellum* GDFS1009 and validamycin A on maize sheath blight

*T. asperellum* GDFS1009, *R. solani*, and *R. zeae* were cultured in a PDA dish for 3 d. Holes were then punched into the dish using 7 mm punchers and turned upside down on the following PDA plates: (1) *R. solani* or *R. zeae* dish on PDA plate containing 500 μg/mL validamycin A; (2) *R. solani* or *R. zeae* dish on PDA plate without validamycin A and separated by 4 cm from *T. asperellum* GDFS1009 dish; (3) *R. solani* or *R. zeae* dish on PDA plate with 500 μg/mL validamycin A and also separated 4 cm from *T. asperellum* GDFS1009 dish; (4) *R. solani* or *R. zeae* dish on PDA plate without validamycin A^25^. The plates were incubated at 28 °C and the inhibition effects were photographed after 1 d and 4 d and quantified with Image J software^26^.

### Detection of growth and competition of *T. asperellum* GDFS1009 treated with validamycin A

*T. asperellum* GDFS1009 was cultured in PDA dishes for 3 d, after which holes were punched in the dish containing *T. asperellum* GDFS1009 culture with 7 mm punchers. The dish was then turned upside down on fresh PDA plate containing 500 μg/mL of validamycin A (or on a control PDA plate without validamycin A). The plates were incubated at 28 °C for 4 d. On day 2, the edge of the colony was cut to observe the mycelium morphology under an optical microscope and also the cellular structure under a transmission electron microscope^27^,^28^. On day 3, the overall status of growth and sporulation of *T. asperellum* GDFS1009 was observed and photographed.

*T. asperellum* GDFS1009 and *F. graminearum* were cultured in a PDA dish for 3 d. Holes were then punched in the dish with 7 mm punchers, after which it was turned upside down on a PDA plate containing 500 μg/mL of validamycin A (or on a control plate without validamycin A). In both cases, the two dishes were 4 cm apart^25^. After incubation at 28 °C for 5 d, competition of *T. asperellum* GDFS1009 with *F. graminearum* was observed and photographed.

### Detection of hyperparasitism of *T. asperellum* GDFS1009 upon treatment with validamycin A

*T. asperellum* GDFS1009 and *F. graminearum* were co-cultured on PDA plates containing 500 μg/mL of validamycin A (or on a control plate without validamycin A, as mentioned above)^25^. After sufficient length of incubation at 28 °C to allow the colonies to contact each other, the part in contact was cut to observe hyperparasitism of *T. asperellum* GDFS1009 under an optical microscope.

In addition, the activities of enzymes chitinase and cellulase, associated with hyperparasitism, were induced in enzyme-inducing medium containing 500 μg/mL validamycin A (control without validamycin) and measured by the 3, 5-dinitrosalicylic acid (DNS) method^29^,^30^,^31^. A GlcNAc standard curve was prepared according to Reissig *et al*.^32^. One unit (U) of the chitinase activity was defined as the amount of enzyme required to release 1 μg of GlcNAc per hour at 37 °C temperature and pH 5.2. A glucose standard curve was established as per the People’s Republic of China Light Industry standard (QB 2583-2003). A unit of enzyme activity was defined as the amount of enzyme needed for production of 1 mg of glucose per milliliter of broth at 50 °C temperature and pH 4.8.

### Detection of global impact of validamycin A on *T. asperellum* GDFS1009 transcription by RNA-sequencing

The spore suspension of *T. asperellum* GDFS1009 was transferred to PD medium containing 500 μg/mL analytical standard validamycin A (HPLC degrade, Kexing, China; control without validamycin A) to a final spore concentration of 10^6^ cfu/mL. After culturing at 28 °C, 180 rpm, for 24 h, the mycelia were collected by vacuum filtration. RNA was extracted and cDNA library was built. Solexa sequencing of single-ends (1 × 50) was then performed that produced 20 million reads per sample. To generate additional data, 48 h samples were also sequenced using Solexa (Single-ends: 1 × 50; 10 million reads).

Conventional analysis was performed on the sequencing data obtained from the transcriptome samples, including data preprocessing, genomic mapping, gene expression analysis, transcript expression analysis, alternative splicing analysis, analysis of differential genes, and GO/KEGG enrichment analysis between differentially expressed genes and nondifferentially expressed genes^33^,^34^. The reference genome for transcriptome analysis was derived from http://genome.jgi.doe.gov/Trias1/Trias1.download.html. Fold change <0.05 represents significantly down-regulated, and fold change >2 represents significantly up-regulated.

Specific analysis about TCA cycle, G protein-MAPK-cAMP signaling pathway was via JGI (Joint Genome Institute) online blast with related reference genes, and the highest homology genes were selected for the expression analysis based RNA-seq data and drawing diagrams^17^,^18^,^19^,^20^,^35^.

Representative genes were validated by qRT-PCR in an ABI 7900 HT Sequence Detection System. The primers used are listed in Supplementary Table S1^17^,^36^. The qRT-PCR reaction system (10 μL) was set up as follows: 5 μL of 2×SYBR Green PCR buffer, 1 μL of 10 μM forward primer, 1 μL of 10 μM reverse primer, 5 ng of cDNA, ddH_2_O up to 10 μL. qRT-PCR amplification conditions were as follows: 50 °C for 2 min; 95 °C for 10 min; 95 °C for 15 s, and 60 °C for 1 min, for a total of 40 cycles. Data analysis was carried out as the method outlined in the Bio-Rad quantitative PCR Application Guide 2^−ΔΔCT^ (Livak).

### Detection of global impact of validamycin A on *T. asperellum* GDFS1009 metabolome by GC-MS analysis

The spore suspension of *T. asperellum* GDFS1009 was transferred to PD medium containing 500 μg/mL validamycin A (or in control medium without validamycin A), to a final spore concentration of 10^6^ cfu/mL. After culturing at 28 °C, 180 rpm, for 24 h, 4 volumes of pre-cooled 60% methanol at −50 °C was added to quench for 20 min, and the mixture was centrifuged at 4 °C at 6,000 rpm for 5 min. The precipitate containing mycelia was collected, washed twice with sterile water at 4 °C and freeze-dried under vacuum till all water was removed. After extraction and derivation, intracellular substances were analyzed by gas chromatography-mass spectrometry (GC-MS) (Agilent 7890 A/5975 C GC-MS). The chromatography conditions were as follows: HP-5MS capillary column (5% phenyl methyl silox: 30 m × 250 μm id, 0.25-μm; Agilent J & W scientific, Folsom, CA); split injection, injection volume of 1 μL, split ratio of 20:1. Inlet temperature was 280 °C; ion source temperature was 250 °C; interface temperature was 150 °C. The temperature program was started at 40 °C for 5 min, raised to 300 °C at 10 °C/min stepwise intervals, and held for 5 min. The carrier gas was helium, with a carrier gas flow rate of 1 mL/min. MS conditions were set as follows: Electrospray ionization (ESI) source, full scan mode, electron energy of 70 eV, quadrupole scan range of m/z 35-780.

Data obtained by comparison and analysis of the information derived from the samples was further analyzed by bioinformatics, which included preprocessing of data, identification of compounds and differential screening of compounds^37^,^38^. Metabolites were annotated based on metabolome databases from the NIST commercial database (NIST 2008) and Wiley 9^39^,^40^. P < 0.05 and VIP > 1 represent significantly changed.

### Detection of degradation and absorption of validamycin A by *T. asperellum* GDFS1009 by LC-MS and GC-TOF-MS

*T. asperellum* GDFS1009 spore suspension was transferred to PD medium containing 500 μg/mL validamycin A to a final spore concentration of 10^6^ cfu/mL. After culturing at 28 °C, 180 rpm, for 7 d, the culture was filtered through a 0.22 μm membrane filter. The filtrate was subjected to LC-MS for detection of validamycin A (PD medium containing 500 μg/mL of validamycin A was used as control) by Waters Quattro Premier XE triple quadrupole mass spectrometer equipped with Ultra Performance Liquid Chromatography (UPLC). Chromatography conditions were: C18 column (1.7 μm, 2.1 × 100 mm), 2 μL injection volume, column temperature 40 °C, automatic feed injector temperature maintained at 4 °C, mobile phase A was ultrapure water, mobile phase B was HPLC grade acetonitrile, mobile phase with 0.1% formic acid (v/v), flow rate was 0.3 mL/min; elution was with linear gradient of mobile phase B: 10% initial for 1 min, 10–90% for 1–6 min, 90% equilibrium for 3 min, 90–10% for 0.5 min after the next sample collection. MS conditions: ESI source, positive and negative ionization mode. Source temperature was 150 °C, desolvation temperature was 350 °C, desolvation gas flow was 500 L/h, cone gas flow was 50 L/h. Positive and negative ion mode ionization capillary voltage was 3.0 kV, sampling cone was 20 eV, extraction cone was 2 eV, quadrupole scan ranged between m/z 100–1500^41^.

The spore suspension of *T. asperellum* GDFS1009 was transferred to PD medium with 500 μg/mL validamycin A at a final spore concentration of 10^8^ cfu/mL. After culturing at 28 °C, 180 rpm, for 4 d, the mycelia of *T. asperellum* GDFS1009 were collected by vacuum filtration and washed twice with 4 °C sterile deionized water. Intracellular substances were extracted (Validamycin A standard was used as positive control), derived and detected using Agilent 7890 GC-TOF-MS equipped with Rxi-5Sil MS capillary column (30 m × 250 μm × 0.25 μm, Restek, USA). Detection conditions were as follows injection volume was 1 μL with non-shunt mode; carrier gas was helium; forward flow velocity was 3 mL/ min; column temperature was 40 °C for 1 h, increased to 330 °C at 10 °C per minute rate, and held for 10 min; forward sampling temperature was 280 °C; transmission line temperature was 280 °C; ion source temperature was 220 °C; ionization voltage was −70 eV; scanning mode was 85–600 m/z; scanning rate was 20 spectra/sec; solvent delay was 366 s^42^.

## Additional Information

**How to cite this article**: Wu, Q. *et al*. Omics for understanding synergistic action of validamycin A and *Trichoderma asperellum* GDFS1009 against maize sheath blight pathogen. *Sci. Rep.* **7**, 40140; doi: 10.1038/srep40140 (2017).

**Publisher's note:** Springer Nature remains neutral with regard to jurisdictional claims in published maps and institutional affiliations.

## Supplementary Material

Supplementary Information

## Figures and Tables

**Table 1:** Impact of validamycin A on detoxification/tolerance mechanism of *T. asperellum* GDFS1009.

JGI gene accession number	Gene function	Fold-change (24 h)	Fold-change (48 h)
fgenesh1_kg.1_#_83_#_Locus8780v1rpkm2.98	Peroxidase	**2.76**	0.76
fgenesh1_pg.9_#_228	Peroxidase	**2.47**	0.95
e_gw1.5.1534.1	Peroxidase	**2.22**	1.15
fgenesh1_pm.12_#_284	Peroxidase	**2.91**	1.92
CE166796_5244	Peroxidase	**2.05**	1.37
e_gw1.4.1409.1	Peroxidase	**2.11**	1.18
fgenesh1_kg.1_#_620_#_Locus2568v1rpkm51.12	Peroxidase	**2.19**	1.69
fgenesh1_pm.17_#_35	Peroxidase	**2.01**	1.06
estExt_Genewise1.C_4_t10187	Monooxygenase	**2.16**	1.26
fgenesh1_pg.18_#_142	Monooxygenase	**2.95**	1.94
estExt_Genewise1Plus.C_12_t20472	Monooxygenase	**2.03**	1.68
CE111627_39204	Monooxygenase	**2.26**	1.22
e_gw1.3.1348.1	Monooxygenase	**2.39**	1.68
e_gw1.7.654.1	Monooxygenase	**2.86**	0.58
fgenesh1_pg.4_#_668	Glutathione S-transferase	**2.28**	1.62
fgenesh1_kg.20_#_27_#_Locus84v1rpkm1084.66	Glutathione S-transferase	**3.09**	**3.74**
estExt_Genewise1Plus.C_180420	Glutathione S-transferase	**2.42**	1.78
fgenesh1_pm.17_#_35	Superoxide dismutase	**2.01**	1.06
fgenesh1_kg.1_#_83_#_Locus8780v1rpkm2.98	Catalase	**2.76**	0.77
fgenesh1_pg.9_#_228	Catalase	**2.47**	0.95
e_gw1.6.1782.1	Thioredoxin	**3.05**	**2.85**
gm1.2461_g	ABC translocator	**2.11**	**2.14**
gm1.4091_g	ABC translocator	**1.82**	**2.19**
fgenesh1_kg.12_#_214_#_Locus2695v1rpkm48.25	ABC translocator	1.97	**2.10**
estExt_fgenesh1_pm.C_5_t10252	ABC translocator	1.09	**2.12**
estExt_Genewise1.C_1_t10054	ABC translocator	**2.22**	0.59
estExt_Genewise1Plus.C_4_t30001	ABC translocator	**2.87**	1.00
estExt_Genemark1.C_8_t10500	ABC translocator	**2.41**	1.30
e_gw1.29.56.1	ABC translocator	**2.03**	***0.40***
estExt_fgenesh1_pm.C_250007	ABC translocator	1.33	***0.49***
fgenesh1_kg.5_#_65_#_Locus4653v1rpkm21.43	ABC translocator	0.52	***0.28***

Bold and underlined font represents up-regulated significantly, while bold and italic font represents down-regulated significantly.
