# Supplementary material for: Omics for understanding synergistic action of validamycin A and Trichoderma asperellum GDFS1009 against maize sheath blight pathogen
Source: Sci Rep. 2017 Jan 6;7:40140. doi: 10.1038/srep40140 (PMC5216365; doi:10.1038/srep40140)
Supplement: Supplementary Information [file srep40140-s1.pdf]

Omics for understanding synergistic action of validamycin A and *Trichoderma asperellum* GDFS1009 against maize sheath blight pathogen

Qiong Wu[1], Lida Zhang[1], Hai Xia[1], Chuanjin Yu[1],

Kaidou[1], Yaqian Li[1], and Jie Chen[1,2,3]*

Supplementary figure legends:

Supplementary Figure S1 Statistical analysis of synergistic effect of validamycin A and *T. asperellum* on sheath blight. a, Synergistic effect on *R. solani*; b, Synergistic effect on *R. zeae*.

Supplementary Figure S2 Global impacts of validamycin A on *T. asperellum* GDFS 1009. a, Impacts on RNA level based on RNA-seq (24 h); b, Impacts on RNA level based on RNA-seq (48 h); c, Impacts on primery metabolism based on GC-MS (24h).

Supplementary Figure S3 Impact of validamycin A on hyperparasitism of *T. asperellum* GDFS 1009. a, The effect of validamycin A on the chitinase activity of *T. asperellum* GDFS1009； b, The effect of validamycin A on the cellulase activity of *T. asperellum* GDFS1009；c, Impact of validamycin A on MAP Kinase gene in G protein-MAPK-cAMP pathway of *T. asperellum* based on qRT-PCR; d, Impact of validamycin A on *ech42*, *nag2* and *bgn13.1* genes in G protein-MAPK-cAMP pathway of *T. asperellum* based on qRT-PCR.

Supplementary tables:

Supplementary Table S1 Primers of genes in G protein-MAPK-cAMP pathway for qRT-PCR.

Supplementary Table S2 Impact of validamycin A on genes in TCA cycle of *T. asperellum* based on RNA-seq.

Supplementary Table S3 Impact of validamycin A on genes of carbohydrate, fatty acid and amino acid metabolism related to TCA cycle of *T. asperellum* based on RNA-seq.

Supplementary Table S4 Impact of validamycin A on compounds in TCA cycle of *T. asperellum* based on GC-MS analysis.

Supplementary Table S5 Impact of validamycin A on amino acid and amine of *T. asperellum* based on GC-MS analysis.

Supplementary Table S6 Impact of validamycin A on fatty acid of *T. asperellum* based on GC-MS analysis.

Supplementary Table S7 Impact of validamycin A on carbohydrate of *T. asperellum* based on GC-MS analysis.

Supplementary Table S8 Impact of validamycin A on genes in G protein-MAPK-cAMP pathway of *T. asperellum* based on RNA-seq.

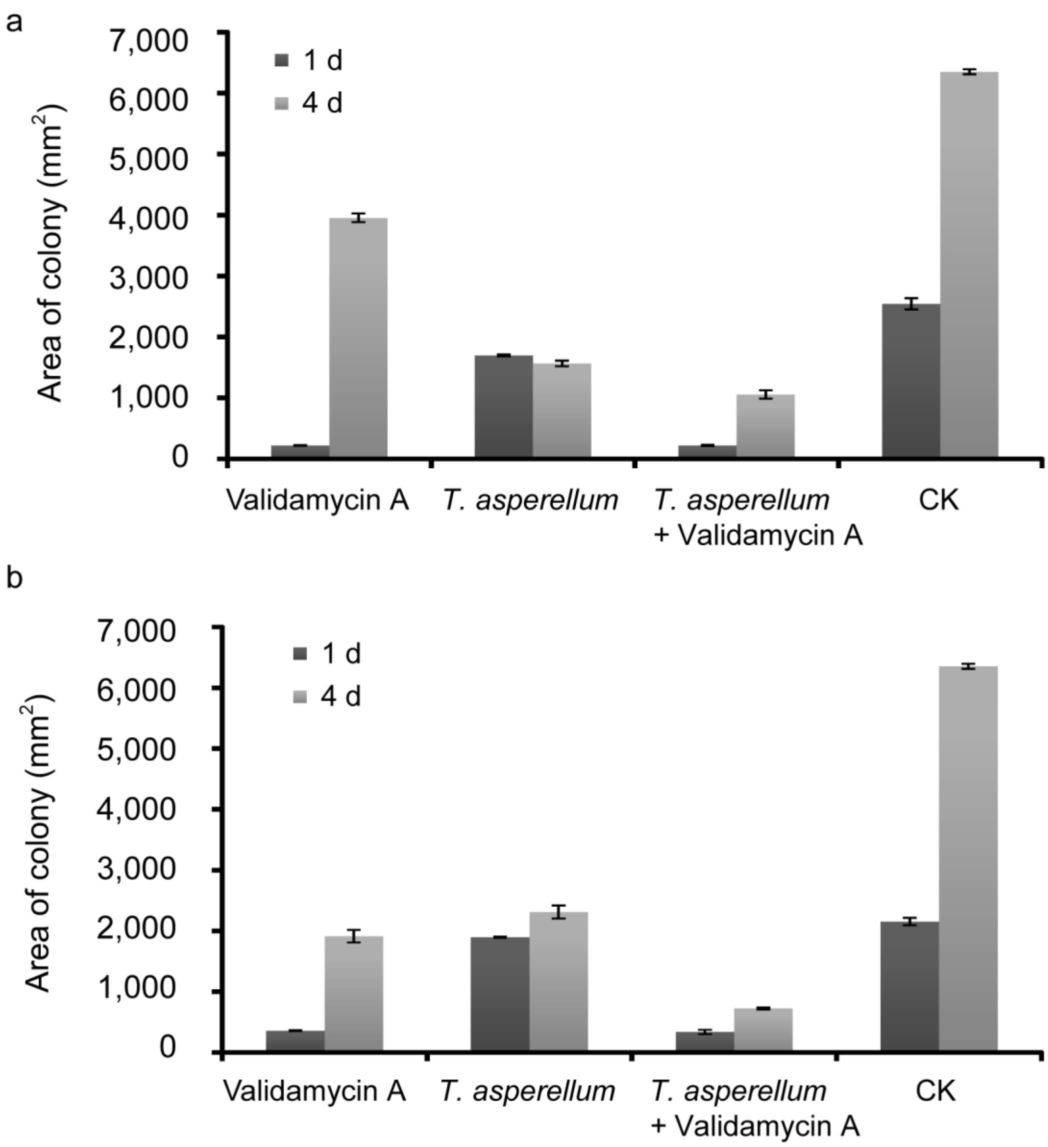

Supplementary Figure S1

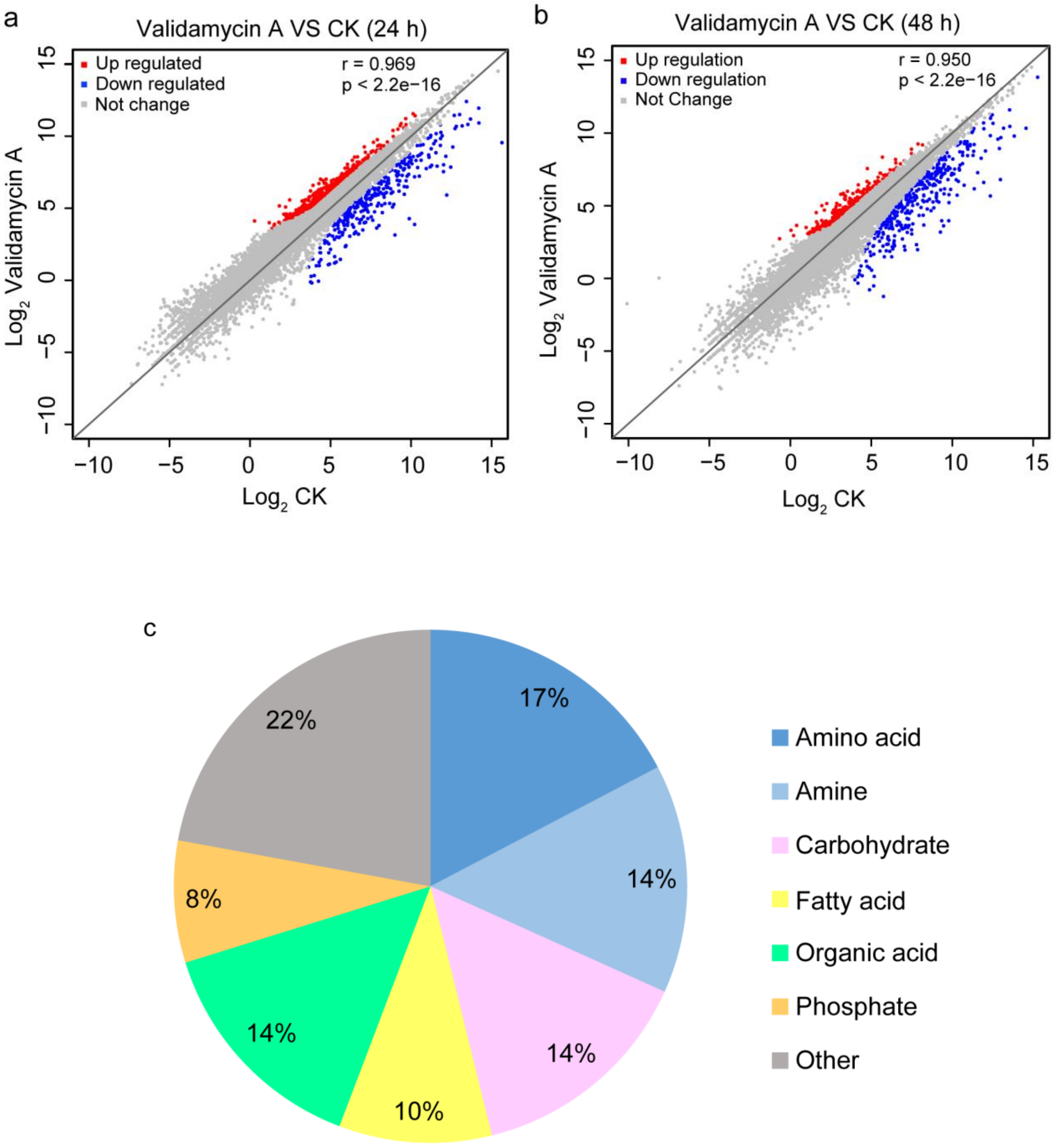

Supplementary Figure S2

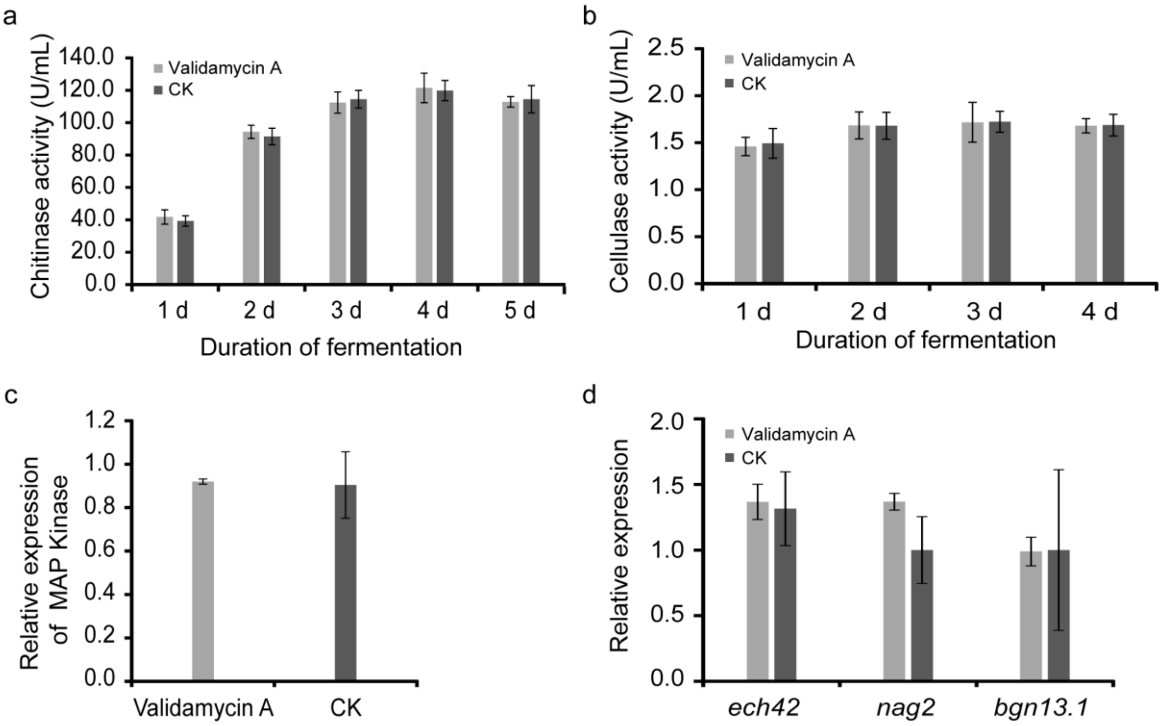

Supplementary Figure S3

Supplementary Table S1

Gene name	Primer name	Primer sequence（5'-3'）
β-actin	1F	GGTATCCACGTCACCACTTTCA
	1R	TGCCGTACAGGTCCTTTCG
MAP Kinase	2F	CAACCCCGTGAAGCGAAT
	2R	CGTGGTAAGGCTCCAGGTATG
chitinase (*ech42*)	4F	GAACGGTATCTGGGACTACAAGGT
	4R	GCCTGTGCGACAGAGTCGTA
Endo-β-N-acetylglucosaminidase (*nag2*)	5F	CAAGAGGACCATTGACGC
	5R	GCTTAGGCAGTGAAGGGAT
β-1, 3-exoglucanase (*bgn13.1*)	6F	GCAGCCCTCAACAAGGTG
	6R	AGAGCCAACGGGAACCAC

Supplementary Table S2

JGI gene accession number	Gene function	Fold-change (24 h)	Fold-change (48 h)
gm1.5866_g	Citrate synthase	1.12	1.00
estExt_fgenesh1_pm.C_10_t10429	Aconitase	**<u>2.27</u>**	1.50
e_gw1.7.1005.1	Isocitrate dehydrogenase	1.50	1.14
fgenesh1_pg.15_#_5	α- oxoglutarate dehydrogenase complex E1	0.95	0.52
fgenesh1_pm.3_#_944	α- oxoglutarate dehydrogenase complex E2	1.36	0.84
fgenesh1_pg.3_#_893	α- oxoglutarate dehydrogenase complex E3	1.31	0.94
estExt_Genewise1Plus.C_3_t10205	Succinyl-CoA synthetase (α)	1.24	0.94
fgenesh1_pm.3_#_112	Succinyl-CoA synthetase (β)	1.36	0.84
e_gw1.1.2022.1	Succinate dehydrogenase	1.58	1.23
e_gw1.9.121.1	Fumarase	1.06	0.83
fgenesh1_pg.5_#_80	Malic dehydrogenase	0.89	0.55

Bold and underlined font represents up-regulated significantly.

Supplementary Table S3

**The TCA cycle related metabolism pathway**	**24 h**		**48 h**	
	**Significantly up-regulated gene numbers**	**Significantly dn-regulated gene numbers**	**Significantly up-regulated gene numbers**	**Significantly dn-regulated gene numbers**
Glycolysis	<u>**4**</u>	***3***	<u>**2**</u>	***3***
Fatty acid degradation	<u>**3**</u>	***1***	0	***1***
Fatty acid elongation	<u>**1**</u>	0	0	0
Fatty acid metabolism	<u>**1**</u>	0	0	***1***
Biosynthesis of unsaturated fatty acids	0	0	0	***1***
Alanine, aspartate and glutamate metabolism	<u>**6**</u>	***2***	<u>**1**</u>	0
Arginine and proline metabolism	<u>**9**</u>	***1***	<u>**4**</u>	0
β-Alanine metabolism	<u>**5**</u>	0	<u>**1**</u>	0
Cysteine and methionine metabolism	<u>**1**</u>	***1***	<u>**5**</u>	0
Glutathione metabolism	<u>**3**</u>	***1***	<u>**3**</u>	***1***
Glycine, serine and threonine metabolism	<u>**4**</u>	0	0	0
Histidine metabolism	<u>**2**</u>	0	<u>**1**</u>	0
Lysine biosynthesis	<u>**5**</u>	***1***	<u>**1**</u>	0
Phenylalanine metabolism	<u>**7**</u>	***1***	<u>**3**</u>	0
Phenylalanine, tyrosine and tryptophan biosynthesis	<u>**5**</u>	***2***	<u>**1**</u>	0
Tryptophan metabolism	<u>**5**</u>	0	<u>**3**</u>	0
Tyrosine metabolism	<u>**7**</u>	***2***	<u>**4**</u>	***1***
Valine, leucine and isoleucine degradation	<u>**13**</u>	0	<u>**1**</u>	0

Bold and underlined font represents up-regulated significantly, while bold and italic font represents down-regulated significantly.

Supplementary Table S4

Compounds	Fold-change (24h)	P-value	VIP
Citric acid	0.91	0.60	0.12
Succinic acid	0.94	0.60	0.19
Malic acid	0.92	0.11	0.22
Tyrosine	0.76	0.21	0.56
Alanine	0.93	0.18	1.24
Leucine	0.88	0.19	1.62
Isoleucine	0.80	0.13	1.70
Glutamic acid	0.52	0.06	2.10
Valine	0.62	0.12	1.64
Threonine	0.59	0.08	1.17
Methionine	0.48	0.17	0.76
Phenylalanine	0.64	0.12	1.98
Palmitic acid	1.03	0.39	0.83
Stearic acid	0.97	0.59	1.39
Glucose	0.93	0.19	1.26

$P < 0.05$ and VIP > 1 represent significantly changed.

Supplementary Table S5

**Compound name**	**Fold change**	**P-value**	**VIP**
Alanine	0.93	0.18	1.24
Glycine	0.86	0.34	0.98
O-Succinylhomoserine	0.61	0.15	0.62
Valine	0.62	0.12	1.64
Leucine	0.88	0.19	1.62
Isoleucine	0.80	0.13	1.70
Serine	0.86	0.09	1.41
Threonine	0.59	0.08	1.17
β-Alanine	0.72	0.01	0.31
Methionine	0.48	0.17	0.76
4-Aminobutyric acid	***0.71***	***0.03***	***1.88***
Proline	0.42	0.03	0.34
Glutamine	0.61	0.05	0.33
Glutamic acid	0.52	0.06	2.10
Phenylalanine	0.64	0.12	1.98
Asparagine	0.38	0.07	1.07
Lysine	0.76	0.01	0.37
Tyrosine	0.76	0.21	0.56
Trifluoroacetamide	1.35	0.52	0.11
N, N-Dimethyl-2-hydroxy-ethanamine	0.92	0.27	2.48
Acetamide	1.00	0.90	0.06
O-Methyl-N, N-dihydroxyl amine	**1.04**	**0.02**	**2.30**
Ethylamin	1.04	0.04	0.95
N-Ethyl-N-vinyl-acetamide	1.01	0.50	0.08
Carbodiimide	0.82	0.27	0.69
Diethylamine	1.07	0.01	0.44
hydroxylamine	1.03	0.47	0.15
Ethanolamine	0.99	0.43	0.34
Cadaverine	0.89	0.03	0.26
Putrescine	0.97	0.26	0.08
2, 5-Diamino valerolactam	0.50	0.05	0.33
Agmatine	0.54	0.05	0.14
N-Acetyl glucosamine	3.97	0.16	4.07

Bold and underlined font represents up-regulated significantly, while bold and italic font represents down-regulated significantly.

Supplementary Table S6

Compound name	Fold change	P-value	VIP
Palmitelaidic acid	1.09	0.49	0.20
Palmitic acid	1.03	0.39	0.83
Heptadecanoic acid	1.12	0.65	0.08
Linoleic acid	1.64	0.11	2.22
α-Glycerophosphorylglcerol	0.99	0.91	0.03
Stearic acid	0.97	0.59	1.39
Oxyoctadecadienoate	0.77	0.03	0.69
Octadecadienoate	0.79	0.20	0.13
Propyl myristate	***0.74***	***0.03***	***1.03***
2-Monooleoylglycerol	1.89	0.15	0.33

Bold and italic font represents down-regulated significantly.

Supplementary Table S7

**Compound name**	**Fold change**	**P-value**	**VIP**
Ribose	0.76	0.43	1.14
Mannose	0.62	0.00	0.92
Fructose	***0.68***	***0.04***	***1.27***
Galactose	0.62	0.06	2.02
Glucose	0.93	0.19	1.26
Glucitol	1.00	0.98	2.00
β-D-Methylglucopyranoside	0.70	0.09	0.37
Sedoheptulose	0.52	0.01	0.47
Mannitol	***0.52***	***0.02***	***1.52***
Sucrose	1.06	0.52	1.78
Laminaribiose	0.64	0.16	1.24
Lactose	0.75	0.36	0.60
Cellotriose	0.81	0.50	1.58
Maltose	1.01	0.96	0.73
Isomaltose	0.69	0.19	0.50

Bold and italic font represents down-regulated significantly.

Supplementary Table S8

JGI gene accession number	Gene function	Abbreviation	Reference gene accession number	Fold-change (24 h)	Fold-change (48 h)
e_gw1.7.1113.1	G protein-coupled receptor	G-protein	ABB89145.2	**<u>2.14</u>**	1.62
gm1.8852_g	adenylate cyclase	AC	EF189190.1	0.66	1.10
fgenesh1_pm.6_#_174	G-protein α subunit subgroup I	α1	AY190117.1	1.42	1.22
e_gw1.2.1919.1	G-protein α subunit subgroup III	α3	AF452097.1	1.62	1.55
e_gw1.11.810.1	G-protein β subunit	β	AY190116.1	1.52	1.34
fgenesh1_pm.1_#_501	G protein γ subunit	γ	AY823297.1	0.91	0.73
gm1.461_g	cAMP-dependentprotein kinases catalytic subunit	PKA-C	XP_013950871.1	1.88	1.21
e_gw1.1.2538.1	cAMP-dependentprotein kinases regulatory subunit	PKA-R	DQ077817.1	1.14	0.73
fgenesh1_pm.11_#_207	mitogen-activated protein kinase kinase kinase	MAPKKK	EGR50365.1	1.24	1.26
fgenesh1_kg.3_#_841_#_Locus880v1rpkm163.01	mitogen-activated protein kinase	TMK1	AF452096.1	1.55	1.95
fgenesh1_pm.14_#_12	chitinase activity	ECH42	AAF19618.1	1.09	0.68
estExt_Genewise1.C_7_t10211	endo-1,3(4)- β-glucanase	BGN13.1	X84085.1	1.27	0.52

Bold and underlined font represents up-regulated significantly.
